# The Impact of Prenatal Vitamin D on Enamel Defects and Tooth Erosion: A Systematic Review

**DOI:** 10.3390/nu15183863

**Published:** 2023-09-05

**Authors:** Gianina Tapalaga, Bogdan Andrei Bumbu, Sandhya Rani Reddy, Sai Diksha Vutukuru, Akhila Nalla, Felix Bratosin, Roxana Manuela Fericean, Catalin Dumitru, Doru Ciprian Crisan, Nicoleta Nicolae, Magda Mihaela Luca

**Affiliations:** 1 Department of Odontotherapy and Endodontics, Faculty of Dental Medicine, “Victor Babes” University of Medicine and Pharmacy Timisoara, Eftimie Murgu Square 2, 300041 Timisoara, Romania; tapalaga.gianina@umft.ro; 2 Department of Dental Medicine, Faculty of Medicine and Pharmacy, University of Oradea, 410073 Oradea, Romania; 3 Department of General Medicine, Prathima Institute of Medical Sciences, Hyderabad 505417, India; reddysandhya094@gmail.com; 4 Department of General Medicine, MNR Medical College, Hyderabad 502285, India; dikshareddy611@gmail.com (S.D.V.); akhila.nalla@gmail.com (A.N.); 5 Department XIII, Discipline of Infectious Diseases, University of Medicine and Pharmacy “Victor Babes” Timisoara, Eftimie Murgu Square 2, 300041 Timisoara, Romania; felix.bratosin@umft.ro (F.B.); manuela.fericean@umft.ro (R.M.F.); 6 Doctoral School, “Victor Babes” University of Medicine and Pharmacy Timisoara, Eftimie Murgu Square 2, 300041 Timisoara, Romania; 7 Department of Obstetrics and Gynecology, University of Medicine and Pharmacy “Victor Babes” Timisoara, Eftimie Murgu Square 2, 300041 Timisoara, Romania; dumitru.catalin@umft.ro (C.D.); crisan.doru@umft.ro (D.C.C.); nicolae.nicoleta@umft.ro (N.N.); 8 Department of Pediatric Dentistry, Faculty of Dental Medicine, “Victor Babes” University of Medicine and Pharmacy Timisoara, Eftimie Murgu Square 2, 300041 Timisoara, Romania; luca.magda@umft.ro

**Keywords:** pregnancy, Vitamin D, dentistry, dental enamel erosion, tooth diseases, tooth discoloration

## Abstract

Prenatal Vitamin D has been suggested to be critical for dental health in children, affecting outcomes including the prevalence of enamel defects and tooth erosion. This systematic review aimed to evaluate the potential impact of prenatal Vitamin D levels on these dental health outcomes. A total of seven studies, involving 6978 participants, were included after a comprehensive search of PubMed, Web of Science, and Scopus from 2013 to June 2023. The average age of mothers varied across studies, with Vitamin D levels or supplementation practices displaying significant variation among the study populations. The age of children at examination ranged from 3.6 to 6.6 years. The analysis demonstrated a diverse association between Vitamin D levels and dental outcomes, with enamel defects reported in 21.1% to 64% of the children and opacities ranging from 36% to 79.5% across studies. Maternal Vitamin D insufficiency was identified as a significant risk factor for enamel defects in one study (OR: 3.55), whereas high prenatal Vitamin D levels indicated a protective effect against Hypomineralized Second Primary Molars (OR: 0.84) and Molar Incisor Hypomineralization (OR: 0.95) in another. Conversely, low Vitamin D levels increased the risk of enamel hypoplasia (OR: 1.29) and dental decay. The maternal and child demographics varied greatly across the studies, and the assessment and prevalence of Vitamin D deficiency or insufficiency were heterogenous. This review illuminates the potential influence of prenatal Vitamin D on dental health in children, underscoring the importance of adequate Vitamin D levels during pregnancy. However, more robust research is required to establish the optimal Vitamin D intake during pregnancy to ensure healthy dental outcomes in children.

## 1. Introduction

The role of nutrition in maintaining oral health is indisputable, with a diverse array of nutrients known to directly or indirectly influence oral health outcomes [1,2]. Among these nutrients, Vitamin D, widely recognized for its fundamental role in bone health, has garnered significant attention for its potential implications in dental health. Several studies have indicated that Vitamin D might play a crucial role in maintaining the health and integrity of tooth structures [3,4].

Vitamin D is essential for calcium and phosphate homeostasis, contributing significantly to the mineralization of bones and teeth. Vitamin D, an essential micronutrient, is both synthesized in our skin in response to sunlight exposure as well as absorbed through dietary sources. Its synthesis begins with the conversion of 7-dehydrocholesterol in the skin to previtamin D3 under UVB radiation, which then spontaneously isomerizes to form Vitamin D3 (cholecalciferol) due to body heat. This cholecalciferol is hydroxylated in the liver to produce 25-hydroxyvitamin D3 (calcidiol), and further converted into the biologically active form, 1,25-dihydroxyvitamin D3 (calcitriol), in the kidneys [5,6]. Consequently, it is evident that Vitamin D, through its action on calcium and phosphate metabolism, may indirectly affect dental health. Similarly, the enamel is susceptible to various defects, collectively termed as enamel hypoplasia. This is primarily a developmental condition that results in thin, incomplete, and inadequately mineralized enamel [7]. Several factors, both genetic and environmental, influence the development and maturation of enamel. Nutritional factors, particularly Vitamin D, are proposed to play a critical role in this process [8].

Tooth erosion, defined as the irreversible loss of dental hard tissues by a chemical process not involving bacteria, is another dental condition that is of considerable concern. It is commonly attributed to dietary acids; however, the role of systemic factors such as nutritional deficiencies is increasingly being recognized [9,10]. Given that Vitamin D is integral to the mineralization process, it is plausible that its deficiency may contribute to the susceptibility of teeth to erosion. Despite these plausible mechanisms linking Vitamin D to dental health, the evidence remains inconclusive and is characterized by considerable heterogeneity. Some studies have indicated a strong association between Vitamin D and dental health [11,12], while others have reported no significant association [13]. This inconsistency in the literature underscores the need for a more systematic investigation of the role of Vitamin D in enamel defects and tooth erosion.

Based on the existing body of research, our primary hypothesis is that there is a significant association between prenatal Vitamin D levels and the prevalence of enamel defects and tooth erosion in children. This systematic review aims to synthesize the available evidence investigating the role of Vitamin D in enamel defects and tooth erosion in children, as well as to critically evaluate the quality of these studies, and provide an evidence-based summary of the potential implications of prenatal Vitamin D deficiency in dental health.

## 2. Materials and Methods

### 2.1. Protocol and Registration

This systematic review was conducted in June 2023 by searching three electronic databases—PubMed, Web of Science, and Scopus—including literature published up until 2023. The search strategy used medical subject headings (MeSH) keywords such as “Vitamin D”, “Enamel Defects”, “Prenatal”, “Pregnancy”, “Tooth Erosion”, “Dental Health”, “Oral Health”, “Nutritional Deficiency”, and “Dental Caries”. The strategy included the following string: “vitamin D” and “oral health” OR “tooth diseases” OR “pregnancy” OR “prenatal” OR “periodontal diseases” OR “dental erosion” OR “hypomineralisation” OR “tooth erosion” OR “biofilm” OR “periodontitis” OR “gingivitis” OR “nutritional deficiency” OR “dental plaque” OR “plaque”.

The review adhered to the Preferred Reporting Items for Systematic Reviews and Meta-Analyses (PRISMA) guidelines [14] and the International Prospective Register of Systematic Reviews (PROSPERO) criteria [15]. A structured and systematic search strategy was utilized to identify relevant scientific papers examining the impact of Vitamin D on enamel defects and tooth erosion. The systematic review was registered on the Open Science Framework (OSF) platform [16] with the registration code https://doi.org/10.17605/OSF.IO/XDYMV (accessed on 16 August 2023).

The main research question aimed to determine the effects of prenatal Vitamin D levels and supplementation on children’s enamel defects and tooth erosion. We sought to explore and address several sub-questions on topics including the association of Vitamin D deficiency with enamel defects and tooth erosion and the role of Vitamin D in the prevention and treatment of these dental conditions.

### 2.2. Eligibility Criteria

The literature search was limited to English-language journal articles. The selection process started with the removal of duplicate entries, followed by a thorough evaluation of each abstract by two independent researchers to assess its relevance to the research questions. The bibliographies of full-text publications were used for cross-referencing. Subsequently, a comprehensive review of each full text was carried out for the remaining articles to ensure that they met the inclusion criteria. In cases where there were differences of opinion during the study selection process, a third author was consulted to make a final decision. The selection process was carried out by the authors while manually following the outlined criteria. Relevant data from the selected articles were manually extracted and managed by the authors.

The inclusion criteria for the systematic review were: (1) studies addressing the impact of Vitamin D on enamel defects and tooth erosion in children after prenatal measurement of Vitamin D levels; (2) clinical outcome measures including but not limited to dental health, oral health, enamel hypomineralization, enamel defects, tooth erosion, and dental caries; and (3) detailed description of the Vitamin D status assessment methods. Conversely, the exclusion criteria were: (1) studies not addressing the effects of Vitamin D on enamel defects and tooth erosion; (2) studies lacking relevant data on clinical outcomes; (3) articles in which the Vitamin D status assessment method was not explicitly described; and (4) in vitro studies, case reports, proceedings, reviews, commentaries, and letters to the editor. We chose studies that assessed enamel defects and tooth erosion in second primary molars as these teeth are commonly used as index teeth in dental research due to their early eruption and susceptibility to dental caries and enamel defects.

### 2.3. Data Collection Process

The initial search yielded a number of 1204 studies, of which a set number were identified as duplicates. After excluding non-relevant papers based on their abstracts, two authors scrutinized the remaining full-text articles for relevance, while a third author performed a triple check. We also collected data on potential confounding factors such as age, sex, race, socioeconomic status, and other relevant factors. We extracted detailed information on the doses of Vitamin D used in each study, including the form of Vitamin D and the dose, frequency, and duration of supplementation.

We used the Quality Assessment Tool for Observational Cohort and Cross-Sectional Studies to evaluate the included articles. Each question within the tool received a score of 1 for “Yes” responses and 0 for “No” and “Other” responses to determine the final performance score. Research with scores from 0 to 4 was labeled as poor-quality, that scoring between 5 and 9 as fair-quality, and that with a score of 10 or above was deemed excellent-quality. To minimize bias and enhance reliability, two researchers independently assessed the quality of the selected articles, and a third researcher was consulted in cases of conflicting opinions.

### 2.4. Risk of Bias

Publication bias was examined by creating a funnel plot, where the standard error of the log odds ratio was plotted against its corresponding log odds ratio. The symmetry of the plot was visually examined and further assessed using Egger’s regression test, with a *p*-value < 0.05 indicating significant publication bias. A sensitivity analysis was also conducted by removing one study at a time and recalculating the pooled odds ratios to evaluate the robustness of the results and to examine the impact of individual studies on the overall effect size.

## 3. Results

### 3.1. Study Characteristics

**Figure 1:**
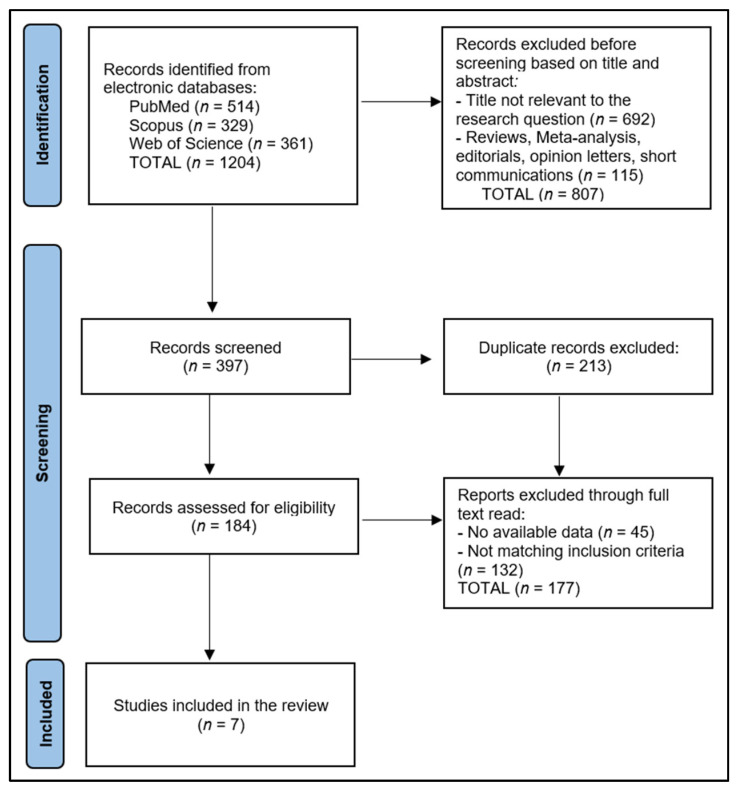
PRISMA Flow Diagram.

The systematic review included seven studies that examined the impact of prenatal Vitamin D on enamel defects and tooth erosion, as presented in Figure 1. These studies were conducted across six countries, namely Denmark, New Zealand, the Netherlands, Norway, and the United States [17,18,19,20,21,22,23], reflecting a broad international interest in this research area. All studies were published from 2014 to 2022, suggesting a continuous and recent exploration into this specific facet of dental health.

The countries of origin for the studies published in 2022 were Denmark, New Zealand, and Norway [17,18,19]. However, the studies conducted in earlier years were distributed across Denmark, the Netherlands, and the USA [20,21,22,23]. This geographical spread indicates global attention being paid to the influence of prenatal Vitamin D on dental health. With regards to study design, the included research either used retrospective cohort studies, prospective cohort studies, or randomized trials, with an approximately equal distribution among these three types. Specifically, two studies used retrospective cohorts [17,18], two used prospective cohorts [21,23], and the remaining three were randomized trials [19,20,22].

The evaluation of study quality showed that half of the studies were characterized as ‘Good’ [17,18,21,23] and the other half as ‘Excellent’ [20,22]. The ‘Good’ quality studies were led by Mortensen et al. [17], Beckett et al. [18], van der Tas et al. [21], and Schroth et al. [23], representing a mix of retrospective and prospective cohort studies. On the other hand, the ‘Excellent’ quality studies, directed by Nørrisgaard et al. [20], Reed et al. [22], and Børsting et al. [19], were all randomized trials, emphasizing the robust design in these investigations. This balance of ‘Good’ and ‘Excellent’ studies suggests a generally high level of quality across the research included in the review (Table 1).

### 3.2. Maternal and Children Characteristics

Table 2 summarizes the maternal characteristics from seven studies investigating the impact of prenatal Vitamin D on enamel defects and tooth erosion. The total number of participants across all studies amounted to 6978, displaying a wide variation in cohort size. The smallest study, conducted by Reed et al. [22], involved only 29 participants, while the most comprehensive study, conducted by van der Tas et al. [21], included a massive cohort of 4750 individuals. The study groups differed across the investigations, including those designated as High-dose Supplementation or Standard-dose Supplementation [20], Sufficient and Insufficient Vitamin D [18], and showing the presence or absence of Hypomineralized Second Primary Molars (HSPM) or enamel hypoplasia [21,22].

The average age of mothers varied across studies, ranging from 19.0 years in the study by Schroth et al. [23] to 32.9 years in Beckett et al.’s study [18]. The assessment of Vitamin D levels or supplementation use was reported in different ways across studies. Supplementation rates ranged from 0.0% in Beckett et al.’s study [18] to 83.7% in Mortensen et al.’s study [17], suggesting diverse Vitamin D supplementation practices among the study populations. The mean or median Vitamin D levels were reported in three of the studies, with values varying from a high of 43.4 ng/mL in Nørrisgaard et al.’s study [20] to the lowest median of 19.2 ng/mL in Schroth et al.’s study [23]. Interestingly, the study by Nørrisgaard et al. [20] provided a direct comparison of Vitamin D levels between high-dose and standard-dose supplementation groups, showing mean Vitamin D levels of 43.4 ng/mL and 28.9 ng/mL, respectively. However, the measures of Vitamin D levels or supplementation were not reported in two studies [18,21], indicating a gap in data availability.

The children’s ages at examination varied from 3.6 years in the study by Reed et al. [22] to 6.6 years in Beckett et al.’s study [18]. Regarding sex distribution, the percentage of females in the studies fluctuated slightly around the 50% mark, indicating a balanced representation of both sexes. The lowest proportion of female participants was 47.1% in Mortensen et al.’s study [17], and the highest was 51.2% in Reed et al.’s study [22], as described in Table 3.

The children’s skin color or race was reported in all studies, indicating the diversity of the cohorts. In most studies, the majority of children were identified as Caucasian, ranging from 44.9% in Reed et al.’s study [22] to 96.9% in Mortensen et al.’s study [17]. Notably, van der Tas et al.’s study [21] and Reed et al.’s study [22] included substantial proportions of Moroccan, Turkish, African, Hispanic, and Black children. Schroth et al.’s study [23] was unique in having a majority of Canadian Aboriginal children, accounting for 90.7% of their cohort.

The assessment of Vitamin D levels was provided in three studies [17,18,21]. Mortensen et al.’s study [17] reported the highest proportion of Vitamin D deficiency, with 58.4% of the children falling into this category, and 16.1% were considered insufficient. Meanwhile, the lowest proportion of Vitamin D deficiency was observed in van der Tas et al.’s study [21], with 26.5% of the children falling into this category and 23.4% identified as insufficient. Beckett et al.’s study [18] showed a more balanced distribution, with 34.6% of the children deficient and 30.9% insufficient. Vitamin D assessment was not reported in the remaining three studies [20,22,23].

### 3.3. Outcomes

**Figure 2:**
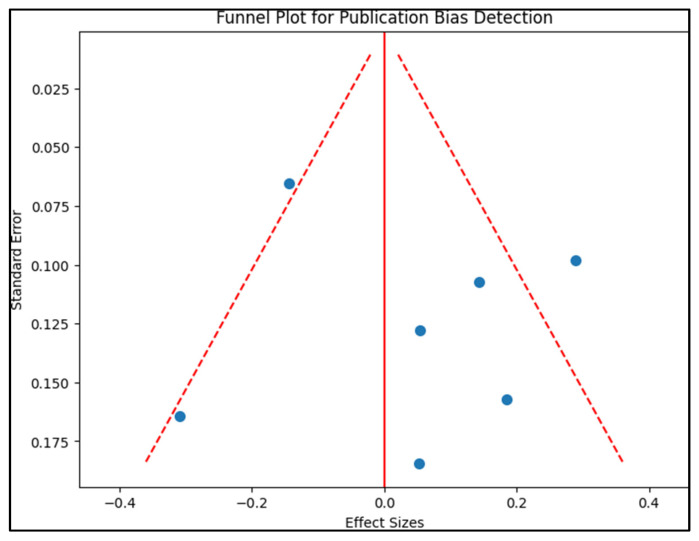
Funnel plot for publication bias.

The results delineated in Table 4 demonstrate varying levels of enamel defects and opacities across the seven studies, with prenatal Vitamin D level suggested as a potential risk factor. The publication bias assessment presented in the funnel plot from Figure 2 indicates the relatively symmetrical distribution of the studies. The study by Mortensen et al. [17] revealed Hypomineralized Second Primary Molars (HSPM) in 54.7% of subjects and opacities in 79.5%. The length of gestation (OR: 0.82) and maternal education (OR: 1.57) were found to be statistically significant factors (*p* < 0.05), implicating that these aspects may have roles in the observed dental anomalies.

In the study by Beckett et al. [18], enamel defects and opacities were reported in 64% and 58% of the participants, respectively. The prevalence of enamel defects was not significantly linked to maternal Vitamin D insufficiency. Still, maternal Vitamin D insufficiency was significantly associated with enamel defects (OR: 3.55), and the insufficiency of Vitamin D in children also carried a risk (OR: 1.64). Nørrisgaard et al. [20] reported enamel defects in 21.1% of the children with deciduous dentition being a statistically significant risk factor (OR: 2.5). There was no difference in the number of erupted permanent molars between the intervention and control groups.

Van der Tas et al. [21] showed HSPM and Molar Incisor Hypomineralization (MIH) in 8.9% and 8.2% of children, respectively. High Vitamin D had a protective effect against HSPM (OR: 0.84) and MIH (OR: 0.95). Children with Vitamin D insufficiency in umbilical cord blood were found to have significantly lower odds of having HSPM. Reed et al. [22] found enamel hypoplasia in 44.8% of their subjects with low Vitamin D slightly increasing the risk (OR: 1.29); however, fetal 25(OH)D concentration was not associated with enamel hypoplasia. Schroth et al. [23] reported enamel hypoplasia in 22% of the examined children, similarly to Børsting et al.’s study, which also reported that 22% of the studied children had HSPM. Opacities were identified in 36% of subjects by Schroth et al. [23] and in 32% by Børsting et al. [19]. Low Vitamin D significantly increased the risk for these outcomes in Schroth’s study (OR: 2.18) and in Børsting’s study (OR: 1.82). Furthermore, a significant inverse relationship was found between the average number of decayed teeth and prenatal 25(OH)D levels.

## 4. Discussion

### 4.1. Summary of Evidence

The current systematic review provides a comprehensive overview of the existing literature, aiming to identify any significant association between prenatal Vitamin D levels and the prevalence of enamel defects and tooth erosion in children. Our review has analyzed seven studies, collectively containing a total of 6978 participants, and observed a variety of outcomes based on varying maternal characteristics, prenatal Vitamin D levels, and postnatal dental assessments.

The findings from our review indicate that prenatal Vitamin D levels can have varying implications on enamel defects and tooth erosion in children. Several studies in our review reported a significant association between prenatal Vitamin D insufficiency and an increased risk of enamel defects [18,19,22,23]. This aligns with our hypothesis and is consistent with the understanding that Vitamin D plays an essential role in calcium metabolism and mineralization processes, which are integral to dental health [24].

Interestingly, the study conducted by van der Tas et al. [21] found a protective effect of high Vitamin D levels against Hypomineralized Second Primary Molars (HSPM) and Molar Incisor Hypomineralization (MIH). This suggests that adequate Vitamin D levels during prenatal development can potentially decrease the risk of these specific dental conditions. These results add to the growing body of literature that underscores the importance of Vitamin D supplementation during pregnancy for the future dental health of the child. Contrary to these findings, Nørrisgaard et al. [20] found no significant difference in the number of erupted permanent molars between their high-dose and standard-dose Vitamin D supplementation groups. This highlights the fact that the relationship between prenatal Vitamin D levels and dental health outcomes in children might not be straightforward and could be influenced by other factors including genetic predisposition, dietary habits, oral hygiene practices, and the presence of other systemic diseases or conditions.

Beckett et al.’s study [18] provided a nuanced perspective by demonstrating a significant association between enamel defects and maternal Vitamin D insufficiency, but no significant link between enamel defects and prenatal Vitamin D insufficiency. This discrepancy indicates the complex nature of Vitamin D’s role in dental health and suggests the necessity for more targeted research to accurately discern the nuances of this association. Moreover, Schroth et al.’s study [23] unveiled an inverse relationship between the average number of decayed teeth and prenatal Vitamin D levels, strengthening the evidence supporting the crucial role of Vitamin D in maintaining oral health. These findings are consistent with previous research indicating that prenatal Vitamin D deficiency might contribute to poor dental health outcomes, including dental caries, in offspring [24,25].

Compared to the risk of enamel defects in prenatal Vitamin D-insufficient mothers and children, there is a certain risk associated with caries as well. One study found a high prevalence of Vitamin D deficiency among pregnant women and children, with 51% of children deficient by age 4 [26]. This deficiency was observed from pregnancy until 8 years of a child’s age, with average 25(OH)D levels falling short of the sufficient level of >30 ng/mL. Additionally, the study noted a tripling in caries risk among children aged 6–10 with 25(OH)D levels <20 ng/mL, matching the levels of their mothers during pregnancy. Finally, factors such as improper brushing techniques and consistent sugar consumption also tripled caries risk.

Another study found no link between the type of toothpaste used and the incidence of caries and enamel defects [27], consistent with one study from Korea that questioned the effectiveness of fluoride in toothpaste and mouthwashes in maintaining anti-acid activity in the mouth [28]. It was also observed that children with occasional sugar consumption had a significantly lower (25%) prevalence of caries compared to those with regular sugar intake, who had more than 60%. This corroborates the general consensus that regular sugar consumption leads to caries, as dietary sugars are converted to acids by oral microorganisms [29,30]. As such, the study recommends using a soft brush from the appearance of the first teeth and limiting sugar intake as essential steps for caries prevention in children.

Another study suggests that Vitamin D deficiency during intrauterine development can cause ameloblast damage and subsequently enamel hypoplasia, a common anomaly in tooth enamel formation [31]. Several studies from various regions align with our findings, both associating Vitamin D deficiency with an increased risk of dental disease in children as well as describing the development of caries. For example, a recent Japanese study of mother–child pairs found a lower risk of dental caries in 3-to-4-year-old children whose mothers had higher Vitamin D intake during pregnancy, although this was based on questionnaires and dietary habits, not directly measured Vitamin D levels [32]. Another recent study from Sweden found an inverse relationship between 25(OH)D levels and caries in 6-year-old children given Vitamin D supplements or a placebo for 3 months [33]. In Canada, preschool children with early caries had lower serum 25(OH)D levels than those without caries [34]. Further, low 25(OH)D levels during pregnancy have been linked to an increased risk of caries, more specifically in the first year of life, although tooth development during the first year of life is minimal or not complete [26]. However, comparing these results worldwide is challenging due to variances in populations, geographical locations, and study designs.

The relationship between prenatal Vitamin D deficiency and Vitamin D levels in later years is indeed complex and not fully understood. It is hypothesized that prenatal Vitamin D deficiency may lead to the altered programming of the Vitamin D metabolism or the immune system in the developing fetus, which could affect Vitamin D levels and immune responses in later life. This may, in turn, impact dental health, as Vitamin D plays a crucial role in maintaining oral health by promoting the mineralization of the tooth enamel and modulating the immune response to oral pathogens. Although our study focused on the effects of prenatal Vitamin D levels and supplementation on enamel defects and tooth erosion in children, it is important to consider that the consequences of prenatal Vitamin D deficiency may extend beyond childhood and have long-lasting effects on oral health throughout life. Further longitudinal studies are needed to investigate the long-term effects of prenatal Vitamin D deficiency on Vitamin D metabolism, immune function, and dental health in adulthood.

### 4.2. Limitations

While our systematic review has yielded important insights into the impact of prenatal Vitamin D on enamel defects and tooth erosion, it is not without limitations. Firstly, there was substantial heterogeneity among the studies included in terms of the study designs, Vitamin D assessment methods, enamel defect assessment, and demographic characteristics of the populations studied. This heterogeneity might limit the comparability of the results across different studies. Secondly, although we collected data on potential confounding factors, we were not able to perform a meta-regression to assess their impact on the results due to the limited number of studies included in the review. Additionally, language bias could have been present as we only included studies published in English.

Moreover, we identified a significant gap in data availability, as some of the studies did not report the measures of Vitamin D levels or supplementation. This deficiency of data might limit the conclusions that can be drawn regarding the association between prenatal Vitamin D and dental health outcomes. Lastly, the cross-sectional nature of the included studies precludes any causal inference about the role of prenatal Vitamin D in the development of enamel defects and tooth erosion. Further prospective and intervention studies are required to confirm the observed associations and to provide more robust evidence for causal relationships.

## 5. Conclusions

In conclusion, our systematic review suggests that prenatal Vitamin D levels and supplementation might have a significant impact on children’s enamel defects and tooth erosion. Thus, adequate prenatal Vitamin D could be an important component of preventive dental health strategies. Nevertheless, due to the significant heterogeneity among the selected studies and the potential impact of confounding factors, further research is needed to confirm these findings. Moreover, the extent and consistency of the observed effects vary between studies, with some showing no association between prenatal Vitamin D and dental outcomes. This variance warrants further research to elucidate the underlying mechanisms and to establish definitive conclusions.

## Figures and Tables

**Table 1:** Study characteristics.

Study & Author	Country	Study Year	Study Design	Study Quality
1 Mortensen et al. [17]	Denmark	2022	Retrospective Cohort	Good
2 Beckett et al. [18]	New Zealand	2022	Retrospective Cohort	Good
3 Nørrisgaard et al. [20]	Denmark	2019	Randomized Trial	Excellent
4 van der Tas et al. [21]	The Netherlands	2018	Prospective Cohort	Good
5 Reed et al. [22]	USA	2017	Randomized Trial	Excellent
6 Schroth et al. [23]	USA	2014	Prospective Cohort	Good
7 Børsting et al. [19]	Norway	2022	Randomized Trial	Excellent

**Table 2:** Maternal characteristics of patients in the included studies.

Study Number	Number of Participants	Study Groups	Average Age (Years)	Vitamin D Assessment
1 Mortensen et al. [17]	1241	HSPM: 679 (54.7%) No-HSPM: 562 (55.3%)	30.6	Supplementation: 83.7% Median: 26.0 ng/mL
2 Beckett et al. [18]	81	Sufficient: 43 (53.1%) Insufficient: 14 (17.3%)	32.9	Supplementation: 0.0% Mean/Median: NR
3 Nørrisgaard et al. [20]	496	High-dose supplementation: 244 (49.2%) Standard-dose supplementation: 252 (50.8%)	32.5	Mean: 43.4 ng/mL vs. 28.9 ng/mL
4 van der Tas et al. [21]	4750	HSPM: 4278 (90.0%) MIH:	30.4	Mean/Median: NR
5 Reed et al. [22]	29	With enamel hypoplasia: 13 (44.8%) Without enamel hypoplasia: 16 (55.2%)	28.6	Mean: 32.1 ng/mL vs. 33.6 ng/mL
6 Schroth et al. [23]	205	Insufficient: 57 (27.8%)	19.0	Mean: 19.2 ng/mL
7 Børsting et al. [19]	176	Gestational week 18–22 vs. Gestational week 32–36	31.2	Mean: 27.4 ng/mL

NR—Not Reported; HSPM—hypomineralized second primary molars; Vitamin D hypovitaminosis (insufficiency) is considered to be below 20 ng/mL or 50 nmol/L; Vitamin D deficiency is considered to be below 10 ng/mL or 25–30 nmol/L.

**Table 3:** Children’s characteristics.

Study Number	Age at Examination	Sex (Female, %)	Skin Color/Race	Vitamin D Assessment
1 Mortensen et al. [17]	4.1 years	47.1%	Caucasian: 96.9%	Deficient: 58.4% Insufficient: 16.1% Median: 18.0 ng/mL
2 Beckett et al. [18]	6.6 years	48.1%	Caucasian: 88.0% Maori: 7.4%	Deficient: 34.6% Insufficient: 30.9%
3 Nørrisgaard et al. [20]	6 years	49.8%	Caucasian: 95.2%	NR
4 van der Tas et al. [21]	6.2 years	50.3%	Caucasian: 65.0% Moroccan and Turkish: 14.0% African: 14.8%	Deficient: 26.5% Insufficient: 23.4%
5 Reed et al. [22]	3.6 years	51.2%	Caucasian: 44.9% Hispanic: 31.0% Black: 24.1%	NR
6 Schroth et al. [23]	6 years	NR	Canadian aboriginal: 90.7%	NR
7 Børsting et al. [19]	8.1 years	48.3%	NR	NR

NR—Not Reported; Vitamin D hypovitaminosis (insufficiency) is considered to be below 20 ng/mL or 50 nmol/L; Vitamin D deficiency is considered to be below 10 ng/mL or 25–30 nmol/L.

**Table 4:** Study outcomes.

Study Number	Outcomes	Risk Assessment (OR)	Other Particularities
1 Mortensen et al. [17]	HSPM: 54.7% Opacities: 79.5%	Length of gestation: 0.82 * Maternal education: 1.57 *	yellow/brown opacities: 14.9%; post-eruptive breakdown: 5.2%; atypical restoration: 0.4%
2 Beckett et al. [18]	Enamel defects: 64% Opacities: 58%	Maternal Vitamin D insufficiency: 3.55 Vitamin D insufficiency in the children: 1.64	yellow/brown opacities: 49.4%; Vitamin D insufficiency was not significantly associated with enamel defect prevalence.
3 Nørrisgaard et al. [20]	Enamel defects: 21.1%	Decidious dentition: 2.5 *	Decidious dentition: 12.3% There was no difference in the number of erupted permanent molars between the intervention and the control group.
4 van der Tas et al. [21]	HSPM: 8.9% MIH: 8.2%	High Vitamin D: 0.84 for HSPM High Vitamin D: 0.95 for MIH	The fetal 25(OH)D concentration was not associated with the presence of MIH in children. Children with Vitamin D insufficiency in umbilical cord blood had significantly lower odds of having HSPM than children with sufficient to optimal levels.
5 Reed et al. [22]	Enamel hypoplasia: 44.8%	Low Vitamin D: 1.29	The fetal 25(OH)D concentration was not associated with the presence of enamel hypoplasia.
6 Schroth et al. [23]	Enamel hypoplasia: 22% Opacities: 36%	Low Vitamin D: 2.18 *	There was a significant inverse relationship between the average number of decayed teeth and prenatal 25(OH)D levels.
7 Børsting et al. [19]	HSPM: 22% MIH: 32%	Maternal Vitamin D insufficiency: 1.82 *	The presence of insufficient maternal Vitamin D levels at mid-pregnancy was related with a larger proportion of MIH in kids at 7–9 years of age.

*—statistically significant values; NR—Not Reported; OR—Odds Ratio; HSPM—hypomineralized second primary molars; MIH—molar incisor hypomineralization.

## Data Availability

Not applicable.

## References

[1] Moynihan P., Petersen P.E. (2004). Diet, nutrition and the prevention of dental diseases. Public Health Nutr..

[2] Tungare S., Paranjpe A.G. (2023). Diet and Nutrition to Prevent Dental Problems. StatPearls [Internet].

[3] Hujoel P.P. (2013). Vitamin D and dental caries in controlled clinical trials: Systematic review and meta-analysis. Nutr. Rev..

[4] Botelho J., Machado V., Proença L., Delgado A.S., Mendes J.J. (2020). Vitamin D Deficiency and Oral Health: A Comprehensive Review. Nutrients.

[5] Holick M.F. (2007). Vitamin D deficiency. N. Engl. J. Med..

[6] Diachkova E., Trifonova D., Morozova E., Runova G., Ashurko I., Ibadulaeva M., Fadeev V., Tarasenko S. (2021). Vitamin D and Its Role in Oral Diseases Development. Scoping Rev. Dent. J..

[7] Suckling G.W. (1989). Developmental defects of enamel-historical and present-day perspectives of their pathogenesis. Adv. Dent. Res..

[8] Wright J.T. (2023). Enamel Phenotypes: Genetic and Environmental Determinants. Genes.

[9] Lussi A., Carvalho T.S. (2014). Erosive tooth wear: A multifactorial condition of growing concern and increasing knowledge. Erosive Tooth Wear.

[10] Johansson A.K., Omar R., Carlsson G.E., Johansson A. (2012). Dental erosion and its growing importance in clinical practice: From past to present. Int. J. Dent..

[11] Madi M., Pavlic V., Mongith Alammar S., Mohammad Alsulaimi L., Shaker Alotaibi R., Mohammed AlOtaibi G., Zakaria O. (2021). The association between vitamin D level and periodontal disease in Saudi population, a preliminary study. Saudi Dent. J..

[12] Chhonkar A., Gupta A., Arya V. (2018). Comparison of Vitamin D Level of Children with Severe Early Childhood Caries and Children with No Caries. Int J Clin Pediatr Dent..

[13] Dudding T., Thomas S.J., Duncan K., Lawlor D.A., Timpson N.J. (2015). Re-Examining the Association between Vitamin D and Childhood Caries. PLoS ONE.

[14] Moher D. (2009). Preferred reporting items for systematic reviews and meta-analyses: The PRISMA statement. PLoS Med..

[15] Schiavo J.H. (2019). PROSPERO: An International Register of Systematic Review Protocols. Med. Ref. Serv. Q..

[16] Foster E.D., Deardorff A. (2017). Open Science Framework (OSF). J. Med. Libr. Assoc..

[17] Mortensen N.B., Haubek D., Dalgård C., Nørgaard S.M., Christoffersen L., Cantio E., Rasmussen A., Möller S., Christesen H.T. (2022). Vitamin D status and tooth enamel hypomineralization are not associated in 4-y-old children: An Odense Child Cohort study. J. Steroid Biochem. Mol. Biol..

[18] Beckett D.M., Broadbent J.M., Loch C., Mahoney E.K., Drummond B.K., Wheeler B.J. (2022). Dental Consequences of Vitamin D Deficiency during Pregnancy and Early Infancy—An Observational Study. Int. J. Environ. Res. Public Health.

[19] Børsting T., Schuller A., van Dommelen P., Stafne S.N., Skeie M.S., Skaare A.B., Mørkved S., Salvesen K.Å., Stunes A.K., Mosti M.P. (2022). Maternal vitamin D status in pregnancy and molar incisor hypomineralisation and hypomineralised second primary molars in the offspring at 7–9 years of age: A longitudinal study. Eur. Arch. Paediatr. Dent..

[20] Nørrisgaard P.E., Haubek D., Kühnisch J., Chawes B.L., Stokholm J., Bønnelykke K., Bisgaard H. (2019). Association of High-Dose Vitamin D Supplementation during Pregnancy with the Risk of Enamel Defects in Offspring: A 6-Year Follow-up of a Randomized Clinical Trial. JAMA Pediatr..

[21] van der Tas J.T., Elfrink M.E.C., Heijboer A.C., Rivadeneira F., Jaddoe V.W.V., Tiemeier H., Schoufour J.D., Moll H.A., Ongkosuwito E.M., Wolvius E.B. (2018). Foetal, neonatal and child vitamin D status and enamel hypomineralization. Community Dent. Oral. Epidemiol..

[22] Reed S.G., Voronca D., Wingate J.S., Murali M., Lawson A.B., Hulsey T.C., Ebeling M.D., Hollis B.W., Wagner C.L. (2017). Prenatal vitamin D and enamel hypoplasia in human primary maxillary central incisors: A pilot study. Pediatr. Dent. J..

[23] Schroth R.J., Lavelle C., Tate R., Bruce S., Billings R.J., Moffatt M.E. (2014). Prenatal vitamin D and dental caries in infants. Pediatrics.

[24] Chen Z., Lv X., Hu W., Qian X., Wu T., Zhu Y. (2021). Vitamin D Status and Its Influence on the Health of Preschool Children in Hangzhou. Front. Public Health.

[25] Brown T., Creed S., Alexander S., Barnard K., Bridges N., Hancock M. (2012). Vitamin D deficiency in children with dental caries—A prevalence study. Arch. Dis. Child..

[26] Suárez-Calleja C., Aza-Morera J., Iglesias-Cabo T., Tardón A. (2021). Vitamin D, pregnancy and caries in children in the INMA-Asturias birth cohort. BMC Pediatr..

[27] Duarte M.B.S., Carvalho V.R., Hilgert L.A., Ribeiro A.P.D., Leal S.C., Takeshita E.M. (2021). Is there an association between dental caries, fluorosis, and molar-incisor hypomineralization?. J. Appl. Oral Sci..

[28] Dang M.H., Jung J.E., Lee D.W., Song K.Y., Jeon J.G. (2016). Recovery of acid production in *Streptococcus mutans* biofilms after short-term fluoride treatment. Caries Res..

[29] Plonka K.A., Pukallus M.L., Barnett A.G., Holcombe T.F., Walsh L.J., Seow W.K. (2013). A longitudinal case-control study of caries development from birth to 36 months. Caries Res..

[30] Philip N., Suneja B., Walsh L.J. (2018). Ecological approaches to dental caries prevention: Paradigm shift or shibboleth?. Caries Res..

[31] Nikiforuk G., Fraser D. (1981). The etiology of enamel hypoplasia: A unifying concept. J. Pediatr..

[32] Tanaka K., Hitsumoto S., Miyake Y., Okubo H., Sasaki S., Miyatake N., Arakawa M. (2015). Higher vitamin D intake during pregnancy is associated with reduced risk of dental caries in young Japanese children. Ann. Epidemiol..

[33] Gyll J., Ridell K., Öhlund I., Karlsland Åkeson P., Johansson I., Lif H.P. (2018). Vitamin D status and dental caries in healthy Swedish children. Nutr. J..

[34] Schroth R.J., Levi J.A., Sellers E.A., Friel J., Kliewer E., Moffatt M.E. (2013). Vitamin D status of children with severe early childhood caries: A case-control study. BMC Pediatr..

